# Sum of peak intensities outperforms peak area integration in iTRAQ protein expression measurement by LC-MS/MS using a TripleTOF 5600+ platform

**DOI:** 10.1042/BSR20190904

**Published:** 2019-06-07

**Authors:** Bastien Burat, Julien Gonzalez, François-Ludovic Sauvage, Hassan Aouad, Hélène Arnion, Emilie Pinault, Pierre Marquet, Marie Essig

**Affiliations:** 1 INSERM, UMR 1248 IPPRITT, University of Limoges, Limoges, France; 2 Department of Pharmacology and Toxicology, CHU Limoges, Limoges, France; 3 Department of Nephrology, Dialysis, and Transplantation, CHU Limoges, Limoges, France; 4 BISCEm, FR CNRS 3503 GEIST, University of Limoges, Limoges, France

**Keywords:** iTRAQ, peak intensities, peak areas

## Abstract

In the field of quantitative proteomics, the Isobaric Tags for Relative and Absolute Quantitation (iTRAQ) technology has demonstrated efficacy for proteome monitoring despite its lack of a consensus for data handling. In the present study, after peptide and protein identification, we compared the widespread quantitation method based on the calculation of MS/MS reporter ion peaks areas ratios (ProteinPilot) to the alternative method based on the calculation of ratios of the sum of peak intensities (jTRAQx [Quant]) and we processed output data with the in-house Customizable iTRAQ Ratios Calculator (CiR-C) algorithm. Quantitation based on peak area ratios displayed no significant linear correlation with Western blot quantitation. In contrast, quantitation based on the sum of peak intensities displayed a significant linear association with Western blot quantitation (non-zero slope; Pearson correlation coefficient test, r = 0.296, *P*=0.010**) with an average bias of 0.087 ± 0.500 and 95% Limits of Agreement from −0.893 to 1.068. We proposed the Mascot-jTRAQx-CiR-C strategy as a simple yet powerful data processing adjunct to the iTRAQ technology.

## Introduction

The recent and already widespread large-scale omics technologies enabled the discovery of unexpected mechanisms in the field of physiology, pathophysiology and pharmacology. These techniques investigate DNA (genomics, epigenetics), mRNAs or microRNAs (transcriptomics), proteins (proteomics), lipids and small molecules (metabolomics). When employed in parallel onto these different targets, large-scale omics techniques help seize the many layers of cell responses to pathophysiological stimuli or to drugs, e.g., regulation and transcription of genes, handling of transcripts and translation into proteins [1]. In pharmacology, beyond the first known drug targets, they are major tools to comprehensively explore all intracellular pathways modified by the drug [2,3]. This enables a better understanding of cellular side effects of drugs.

A number of MS-based high-throughput proteomics or ‘shotgun proteomics’ technologies are compatible with relative protein quantitation and offer variable performances in terms of proteome and sequence coverage, precision, accuracy and reproducibility of quantitation or versatility of sample application [4]. Using Label-free Quantification (LFQ), either protein abundance correlates with the measure of peptide precursor ion MS signal intensities or is obtained from the counting of peptide fragment ion MS/MS spectra (spectral counting) [5]. Isotope-coded Affinity Tags (ICAT) was the first labeling technique, which was based on biased protein labeling through tagging of the non-universal residue cysteine with heavy or light tags [6]. Stable Isotope Labeling of Amino Acids in Cell Culture (SILAC) is one of the proteomic approaches using a metabolic labeling based on the introduction of heavy or light amino acids during protein biosynthesis [7]; hence, a high-level reliability and robustness in terms of labeling stability, precision and accuracy [8]. Isobaric Tags for Relative and Absolute Quantitation (iTRAQ) was the first chemical labeling technique (before Tandem Mass Tag [TMT] and the mTRAQ variant) developed to multiplex comparison between protein sets issued from different biological samples, as obtained from a single tandem mass spectrometry run. In iTRAQ, digested peptides, from up to eight different conditions, are labeled by isobaric tags. This allows characterizing peptides, in a condition-independent way, using the first mass spectrometry filter (MS mode) and measuring their relative abundance between the different conditions using the second stage of the tandem mass spectrometer (MS/MS mode) [9], upon the detection of the condition-specific reporter ions of distinct masses in the low mass-to-charge ratio (m/z) region of the MS/MS spectrum.

Although extended multiplexing is the major advantage of iTRAQ, its use benefits from: high reproducibility, precision and accuracy compared with LFQ (like all stable isotope labeling versus LFQ), to the cost of a wider dynamic range, better proteome coverage and faster sample preparation and analysis [10,11]; better sensitivity and proteome coverage compared with ICAT [12]; wide sample applicability, faster sample processing and better proteome coverage compared with SILAC [13]; as well as a valuable ‘toolbox’ that has been built over the past decade thanks to the literature addressing technique drawbacks and methodological solutions to overcome them [14].

The best method to quantify the isobaric tags, hence the relative peptide abundances, is still under scrutiny. The commercially available software used the ratios of peak areas (RPA) based on the initial description stating that the abundance of a collision-released mass reporter ion appeared to be proportional to the trapezoidal integration of peaks at the theoretical mass-to-charge ratio (m/z). Alternatively, the ratios of sum of peak intensities (RPSI) were shown to result in higher sensitivity and more reliable quantitation [15–17]. In this case, the abundance of a mass reporter ion is directly related to its ion counts – height of the peaks – at the theoretical m/z. To the best of our knowledge, performances of the two methodologies (RPA and ratios of the sum of peak intensities [RSPI]) have never been studied and compared in the light of classical molecular biology approaches for protein quantitation (e.g. Western blot).

In iTRAQ, a correct peptide and protein quantitation needs a correct interpretation of MS/MS spectra, which depends on a trustworthy peptide and protein identification. The identification and quantitation can invariably be performed by commercial built-in algorithms integrated to companion software packages; they fully retreat data generated by the manufacturer’s mass spectrometers (e.g. Paragon – ProteinPilot for ABSciex mass spectrometers). Alternatively, analyses may be split into separate stages through a composite suite of commercial or free algorithms integrated to manufacturer-independent software (e.g. Mascot in Mascot Server, jTRAQx). It is noteworthy that, although tools to compute RPA are widely available, the computation of the ratios of RSPI is not supported by any available companion software, and so, it requires an alternative suite.

The first aim of our work was to compare these two strategies of quantitation (RPA and RSPI) to the classical Western blot technique, commonly used as a non-MS validation technique for iTRAQ-based quantitation. The second aim was to further develop an all-in-one protocol, from sample preparation to result reporting, based on the best strategy of quantitation followed by in-house data processing. The present study was carried out on the respective effects of the CalciNeurin inhibitors (CNI) Cyclosporine A (CsA) and Tacrolimus (Tac, a.k.a. FK506) on renal proximal tubular cells, used as a study model. iTRAQ was combined to nano-scale liquid chromatography online with Q-Q-TOF tandem mass spectrometry on proximal tubular cells to investigate whether CsA and Tac nephrotoxicity results from the inhibition of calcineurin or from the modulation of other intracellular pathways targeted by immunophilins.

In this work, we validated the RSPI methodology and we built an automated data processing algorithm called Customizable iTRAQ Ratio Calculator (CiR-C) to refine critical parameters related to peptide confidence and selection. The composite suite made up of the Mascot algorithm, for peptide and protein identification, end-to-end with the jTRAQx software for computation of RSPI at the peptide level and the CiR-C algorithm for data integration and definitive protein quantitation turned out to be a successful combination. It has provided a great improvement compared with already available solutions for iTRAQ-based high-throughput quantitative proteomics and multiplexed analysis of biological systems.

## Experimental methods

### Materials and chemicals

Dulbecco’s Modified Eagle’s Medium-Ham’s F12 (1:1, #31331, Gibco), Fetal Bovine Serum (#10500), 1 M HEPES (#15630), 7.5% Sodium bicarbonate (#25080), 10000 UI.ml^−1^ Penicillin/Streptomycin (#15140), Dulbecco’s Phosphate Buffer Saline (#14190) were purchased from Gibco. Sodium selenite (S5261), CsA (#30024), Tac (F4679), insulin (I4011), triiodothyronine (T6397) and dexamethasone (D4902) were purchased from Sigma–Aldrich. Primary antibodies against porcine Cyclophilin A (ab41984, 1:1000) was purchased from Abcam, anti-β-Actin (MA1-91399, 1:10000), anti-Na+/K+ ATPase α subunit 1 (MA3-929, 1:2000), anti-Cofilin-1 (PA1-24931, 1:10000) and anti-Galectin-1 (#437400, 1:500) were purchased from ThermoFisher. Secondary antibodies were purchased from Sigma–Aldrich (Anti-Mouse IgG [whole molecule]-Peroxidase antibody produced in rabbit, A9044, 1:10,000; Anti-Rabbit IgG [whole molecule]-Peroxidase antibody produced in goat, A9169, 1:10,000) and ThermoFisher (F[ab’]2-Goat anti-Rabbit IgG [H+L] Secondary Antibody, HRP, A24531, 1:10000).

### Cell culture conditions and drug exposure

LLC PK-1 (Lilly Laboratories Porcine Kidney-1) porcine proximal tubule cells (ATCC-CL-101, ATCC, Manassas, VA) were expanded in 75 cm^2^ flasks at 37°C with 5% CO_2_ and passed once confluence was reached. Culture medium consisted in a 1:1 Dulbecco’s Modified Eagle’s Medium-Ham’s F12 mix supplemented with 5% FBS, 15 mM HEPES, 0.1% Sodium bicarbonate, 100 UI.ml^−1^ Penicillin/Streptomycin and 50 nM Sodium selenite. LLC PK-1 cells were cultured between passage 7 and passage 25.

LLC PK-1 were seeded in four 60 mm Petri dishes (one per condition) and expanded up to sub-confluence in the routine cell culture medium.

Seeded LLC PK-1 sustained serum starvation and were fed with hormonally defined (25 µg.ml^−1^ insulin, 11 µg.ml^−1^ transferrin, 50 nM triiodothyronine, 0.1 µM dexamethasone, 0.1 µg.ml^−1^ desmopressin) fresh medium to engage epithelial differentiation, for 24 h.

Differentiated LLC PK-1 cells were exposed for 24 h to four different conditions: Control (vehicle: 96% Ethanol), 5 µM CsA, 0.05 µM Tac or 1 µM VIVIT (a specific Nuclear Factor of Activated T cells [NFAT] inhibitor [18]).

### Protein extraction, sample preparation, iTRAQ labeling and isoelectric focusing

After 24-h drug exposure, LLC PK-1 cells were washed twice with Dulbecco’s Phosphate Buffer Saline and lysed by scrapping in a custom RIPA lysis buffer (150 mM NaCl, 50 mM TRIS-HCl, 0.1% NP-40, 0.1% SDS, 1 mM EDTA in ultrapure H_2_O, supplemented with an anti-protease/anti-phosphatase mix). Cell lysates were incubated on ice for 30 min and centrifuged for 15 min at 21000 ***g***. Supernatants were stored until protein concentration was measured using the Bradford colorimetric method and iTRAQ labeling. Twenty-five micrograms of proteins were precipitated by −20°C cold acetone. After acetone evaporation, the precipitates were solubilized in 25 mM ammonium bicarbonate then were incubated with 50 mM dithiothreitol for 40 min at 60°C, to reduce disulfide bonds, 100 mM iodoacetamide in the dark for 40 min at room temperature, to alkylate/block cysteine residues and eventually were digested for 24 h at 37°C with mass-spectrometry grade trypsin (V5280, Promega) at a 1:50 enzyme:substrate ratio.

**Scheme 1:**
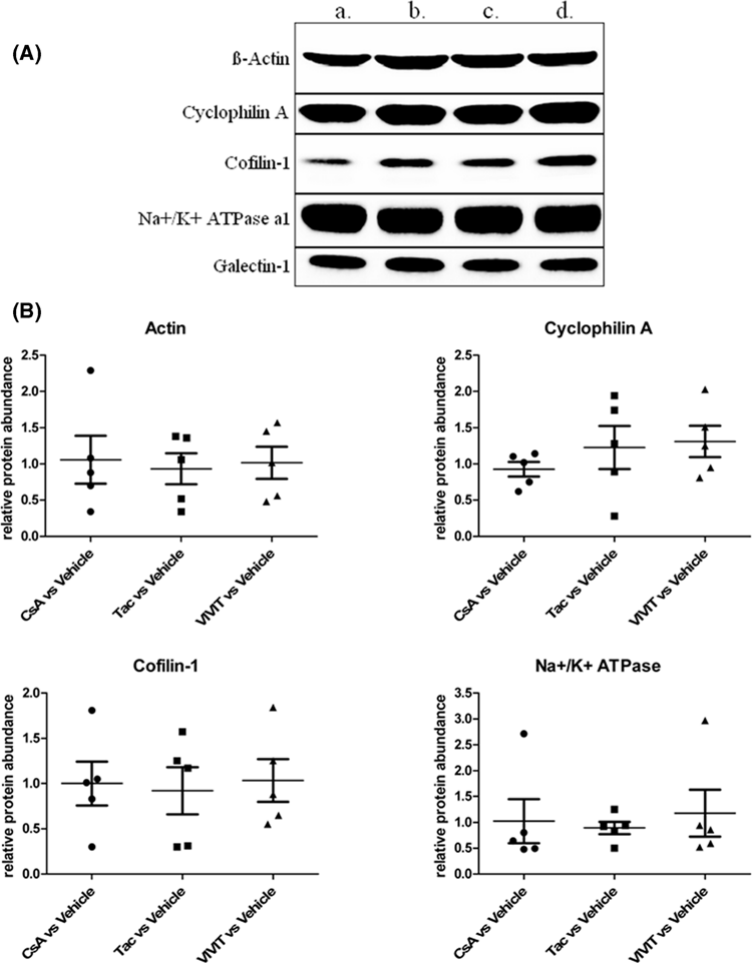
Introduction to iTRAQ technology (**A**) Protocol walk-through. (**B**) Theoretical comparison between strategies of MS/MS peak integration, RPA and RSPI.

After digestion, samples were incubated with iTRAQ tags (iTRAQ Reagents Multiplex kits, 4-plex, #4352135, Sigma–Aldrich) – one tag per drug exposure condition for 1 h at room temperature – and then mixed together. The tags were interchanged between the five independent experiments (biological replicates) to circumvent tag-related bias (Table 1). Mixed labeled samples were separated into 12 fractions by isoelectric focusing (OFFGEL 3100 Fractionator, Agilent Technologies, Santa Clara, CA) for 24 h at increasing voltage and steady intensity of 50 µA in a 3–10 pH IPG strip. Fractions were retrieved for further MS analysis after the IPG strip was incubated in a 1:1 acetonitrile (ACN): water, 0.1% formic acid (FA) wash solution for 15 min at room temperature (Scheme 1A).

**Table 1:** Summary of experimental design

Sample	Set	Condition	4-plex distribution	System	Database
LLC PK-1	1	Control	114	DIONEX UltiMate^®^ 3000 nano-HPLC online with ABSciex 5600+ TripleTOF™ high-resolution mass spectrometer	*Sus scrofa* SwissProt 2015_01 Trembl 2015_01
		CsA	115		
		Tac	116		
		VIVIT	117		
	2	Control	115		
		CsA	116		
		Tac	117		
		VIVIT	114		
	3	Control	116		
		CsA	117		
		Tac	114		
		VIVIT	115		
	4	Control	114		
		CsA	115		
		Tac	116		
		VIVIT	117		
	5	Control	114		
		CsA	115		
		Tac	116		
		VIVIT	117		

Proteins from five independent replicates of four distinct experimental conditions were extracted from LLC PK-1 cells. Peptides were labeled with iTRAQ 4-plex reagents and analyzed by nano-HPLC MS/MS. The 4-plex distribution rotated between independent experiments (biological replicates) to circumvent tag-related bias.

### nano-LC peptide separation and Q-Q-TOF mass spectrometry

IEF fractions were analyzed by nano-LC–MS/MS using a nano-chromatography liquid Ultimate 3000 system (LC Packings DIONEX, Sunnyvale, CA) coupled to a Triple TOF 5600+ mass spectrometer (ABSciex, Toronto, Canada). For each sample, 5 µl were injected into a pre-column (C18 Pepmap™ 300 µm ID × 5 mm, LC Packings DIONEX) using the loading unit. After desalting for 3 min with loading solvent (2% ACN, 0.05% trifluoroacetic acid [TFA]), the pre-column was switched online with the analytical column (C18 Pepmap™ 75 µm ID × 150 mm, LC Packings DIONEX) pre-equilibrated with 95% solvent A (ACN 5% – FA 0.1%). Peptides were eluted from the pre-column into the analytical column and then into the mass spectrometer by a linear gradient from 5 to 25% in 70 min, then to 95% of solvent B (98% ACN, 0.1% FA) over 120 min at a flow rate of 200 nl/min.

Data-Dependent Acquisition (DDA) was carried out by IDA (Information-Dependent Acquisition) mode of Analyst 1.7 TF software (ABSciex). The data from MS and MS/MS were continuously recorded with a cyclic duration of 3 s. For each MS scan, up to 50 precursors were selected for fragmentation based on their intensity (greater than 20,000 cps), their charge state (2+ and 3+) and if the precursor had already been selected for fragmentation (dynamic exclusion). The collision energies were automatically adjusted according to charge state, ionic mass of selected precursors and iTRAQ labeling.

### Mass spectrometry data processing and relative protein identification/quantification

### Quantitation method based on RSPI

MS and MS/MS data for five independent experiments (biological replicates) (*.wiff, 1 per fraction, 12 files per experiment) were submitted to Mascot Server 2.2.03 via ProteinPilot (version 5.0, ABSciex) for protein identification, and searched against two complementary *Sus scrofa* databases: a Swiss-Prot database (2015_10 release) and a TrEMBL database (2015_10 release). Carbamidomethyl (C) was defined as a fixed modification. Oxidation (O), iTRAQ4plex (K), iTRAQ4plex (Y), iTRAQ4plex (N-term) were defined as variable modifications. MS/MS fragment mass tolerance was set at 0.3 Da. Precursor mass tolerance was set at 0.2 Da.

Mascot raw data files (*.dat, 1 per experiment) were saved for further isobaric tags-based peptide and protein quantitation with the Java implementation of the Quant algorithm, jTRAQx (version 1.13, [19]). Reporter mass tolerance was set at 0.05 Da while iTRAQ correction factors were implemented as provided by ABSciex. This tool generated one .jpf file (tab-delimited text file) for each series.

### Quantitation method based on RPA

MS and MS/MS data for five independent experiments (biological replicates) (*.wiff, 1 per fraction, 12 files per experiment) were submitted to protein identification using the Paragon algorithm as implemented in the ProteinPilot software (version 5.0, AB SCIEX) and searched against two complementary *Sus scrofa* databases: a Swiss-Prot database (2015_10 release, 1422 entries) and a TrEMBL database (2015_10 release, 47465 entries). Quantitation was conducted with or without auto bias correction, an available option implemented in the ProteinPilot software to normalize uneven protein across the multiplex samples, improving further quantitation. Mass tolerances and identification parameters were automatically set and optimized for the ABSciex 5600+ TripleTOF™-generated MS/MS data (MS/MS Fragment mass tolerance was set at 0.1 Da. Precursor mass tolerance was set at 0.05 Da).

The *.group results files (1 per experiment) were exported as Peptide Summaries.

### Preparation of spiked-in proteins extracts for iTRAQ benchmarking

The Universal Protein Standard mixture 1 (UPS1, Sigma–Aldrich) containing 48 different human proteins was spiked into a 25 µg protein extract from a control LLC-PK-1 lysate in three UPS1:LLC PK-1 proteins ratios (1:20, 1:15 and 1:25) corresponding to a spike-in of 500 ng (reference UPS1 protein abundance), 625 ng (25% increase in UPS1 protein abundance) and 375 ng (25% decrease in UPS1 protein abundance) UPS1 proteins. Spiked-in protein extracts were prepared in three independent experiments (biological triplicates) and processed as detailed above.

### Western blot

Western blots were performed on total cell lysates from five independent experiments (biological replicates), prepared in custom RIPA buffer (see above). Forty micrograms of proteins per exposure condition were separated by electrophoresis under reducing and denaturing conditions on a NuPAGE^®^ Novex^®^ Bis-Tris pre-cast gel (NP0341, ThermoFisher) in 1X NuPAGE™ MOPS SDS running buffer (NP0001, ThermoFisher) and transferred to a nitrocellulose (NC) membrane (NP23001, ThermoFisher) using the iBLOT 2 Dry Blotting system (IB21001, ThermoFisher). Membranes were blocked for 1 h at room temperature under agitation with TBS-Tween buffer (10 mM Tris 7.6, 150 mM NaCl, 0.1% Tween-20) complemented with 5% (W/V) non-fat milk powder to obtain BLOTTO buffer. Primary antibody incubation was done in BLOTTO for 1 h at room temperature. After three 5-min washes in TBS-T, secondary antibody incubation was performed in BLOTTO for 1 h at room temperature then washed again. Membranes were incubated in a 1:1 mix of SuperSignal™ West Pico PLUS Chemiluminescent Substrate kit (#34577, ThermoFisher) and analyzed by the ChemiDoc Imaging system (Bio-Rad) for chemiluminescent signal detection and acquisition. Quantitation was computed via the ImageLab software (Bio-Rad).

### Statistical analysis

Western blot quantitation was compared with RSPI- and RPA-based quantitation using the Bland–Altman comparison method [20,21] and Pearson correlation coefficient test (minimum significance threshold *P*=0.05). iTRAQ ratios of UPS1 spiked-in proteins were compared using one-way ANOVA analysis and Dunnett’s post-tests (minimum significance threshold *P*=0.05). Statistical analysis was performed using available tests as implemented in the GraphPad Prism software (version 5.04). Protein ratios were calculated as median values of peptide ratios. No further data transformation or data normalization was performed prior to statistical analysis.

### Data availability

The MS proteomics data have been deposited to the ProteomeXchange Consortium (http://proteomecentral.proteomexchange.org) [22] via the PRIDE partner repository [23] with the dataset identifier PXD007891 (username: reviewer72095@ebi.ac.uk, password: TPSGICw9).

## Results

### Comparison between the data processing outputs: MASCOT – jTRAQx versus Paragon – ProteinPilot

Multiplex analysis of CNI-exposed partial proteomes was conducted and optimized *in vitro* using the epithelial tubular proximal cell line LLC PK-1 serving as a model. Five series of samples were prepared and processed according to the optimized custom-made iTRAQ protocols (Scheme 1A). The samples labeling strategy is summarized in Table 1.

The RPA strategy resulted in Paragon identifying 9788 ± 3270 peptides related to 1100 ± 132 proteins per series. After identification and quantitation by the Paragon – ProteinPilot suite, the CiR-C algorithm included 4105 peptides (114 proteins) split into 35 Swiss-Prot entries and 79 TrEMBL entries.

The RSPI strategy resulted in Mascot Server identifying 14291 ± 4582 trypsin-digested peptides related to 1160 ± 115 proteins per series. After identification by the Mascot – jTRAQx suite, the CiR-C algorithm included 32169 peptides (370 proteins) split into 131 Swiss-Prot and 239 TrEMBL entries.

The CiR-C shell script excluded irrelevant data according to criteria summed up in Table 2: (i) identification confidence: peptides are retained if the probability that the observed positive match is a random match is below 5% (*P*<0.05, Mascot score > 30; Paragon confidence score > 95); (ii) quantification confidence: peptides are retained if all iTRAQ ratios have been successfully calculated, i.e., peptides with 0.0 ratios or uncalculated ratios (null ratios) are discarded; (iii) peptides related to ‘Fragment’- and ‘REVERSED’-annotated proteins are discarded. After irrelevant data removal, CiR-C drew up an exhaustive catalog of identified peptide sequences with their associated Swiss-Prot or TrEMBL accession IDs. Peptides were assigned to a frequency index of positive matches (identification in {1;2;3;4;5} out of 5 independent experiments [biological replicates]) and CiR-C drew a second catalog of peptides with the highest frequency index (*n*=5). Protein ratios were calculated as both overall and series-specific median values of peptide ratios associated with a given accession ID and frequency index.

**Table 2:** Data processing summary

Set	Quantitation method	Identified peptides	Identified proteins	Exclusion criteria	Discarded peptides	Retained peptides	Retained proteins
1		6676	943	Confidence score < 95	4626 (69%)		
2	RPA	8926	1044	Null ratio	6263 (70%)		
3	Paragon + Protein Pilot	7269	1040	‘REVERSED’ annotation	4962 (68%)	4105	114
4		11465	1227	‘Fragment’ annotation	10143 (88%)		
5		14604	1247	Shared peptides	11990 (82%)		
1		10984	1086	Mascot score <30	2808 (26%)		
2	RSPI	12379	1028	Null ratio	3114 (25%)		
3	Mascot + jTRAQx	11844	1164	‘Fragment’ annotation	3051 (26%)	32169	370
4		14000	1192	Shared peptides	4463 (32%)		
5		22247	1331		8450 (38%)		
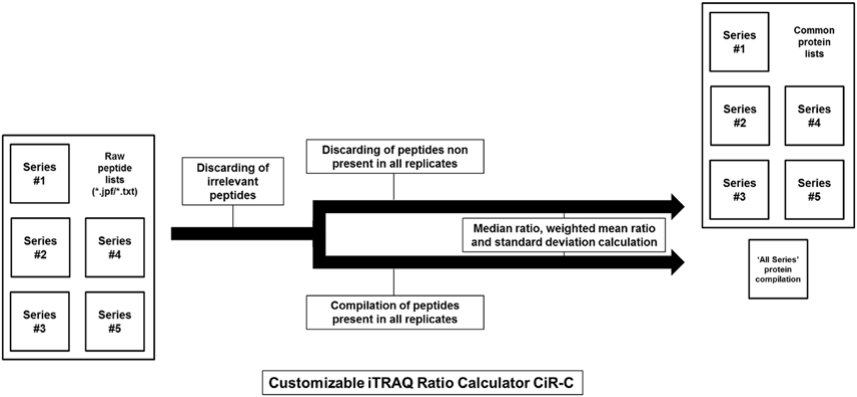							

The MS/MS data from the five replicate experiments were submitted to identification (Mascot versus Paragon), primary quantification (jTRAQx versus ProteinPilot) and data refining, statistical analysis (CiR-C). This table sums up data inclusion criteria, identification output and data refining yield, as numbers of identified, discarded of retained peptides

CiR-C discarded 7597 ± 3291 (75.4% ± 9.0) peptides quantified by RPA and 4377 ± 2367 (29.4% ± 5.5) peptides quantified by the RSPI (Table 2).

### Comparison between the two quantitation strategies

**Figure 1:**
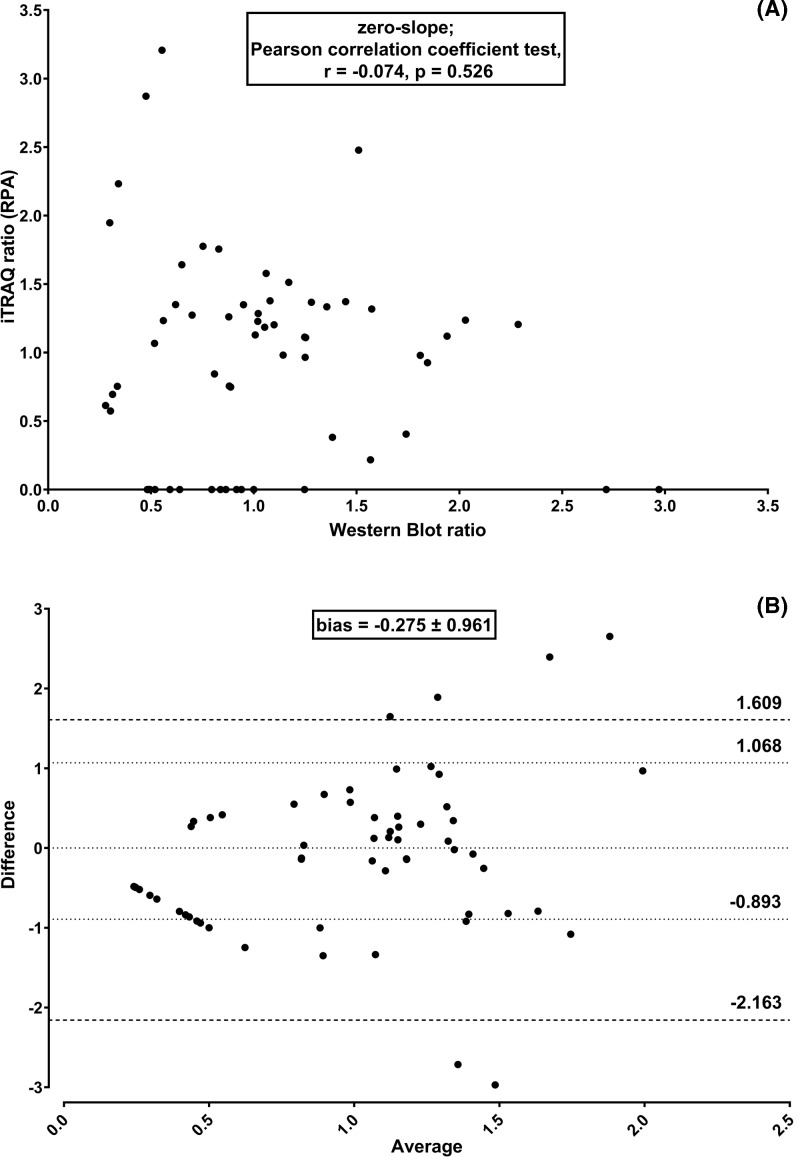
Western blot analysis of five iTRAQ-quantified proteins for quality control and comparison of iTRAQ quantitation strategies (**A**) Representative Western blot of β-Actin, Cyclophilin A, Cofilin-1, Na+/K+ ATPase subunit α-1, and Galectin-1 after 24-h exposure to : (a) Vehicle, (b) CsA 5 µM, (c) Tac 0.05 µM, (d) VIVIT 1 µM. (**B**) Scatter plots of Western blot ratios and relative protein abundance of β-Actin, Cyclophilin A, Cofilin-1, Na+/K+ ATPase subunit α-1, normalized to Galectin-1, expressed as mean ± S.E.M.

The results of the two quantitation strategies (RPA from the Paragon – ProteinPilot – CiR-C data processing and RSPI from the Mascot Server – jTRAQx – CiR-C data processing, Scheme 1B) were compared with the Western blot analysis of five proteins picked from the 370-protein final list (Figure 1A). The selection comprised the cytoskeleton-structuring β-Actin, cytoplasmic Cofilin-1, α-1 subunit of membrane-attached Na^+^–K^+^ ATPase, cytosolic Cyclosporine-complexing Cyclophilin A and Galectin-1, i.e., a panel of both spatially scattered and differently expressed proteins (Figure 1B).

**Figure 2:**
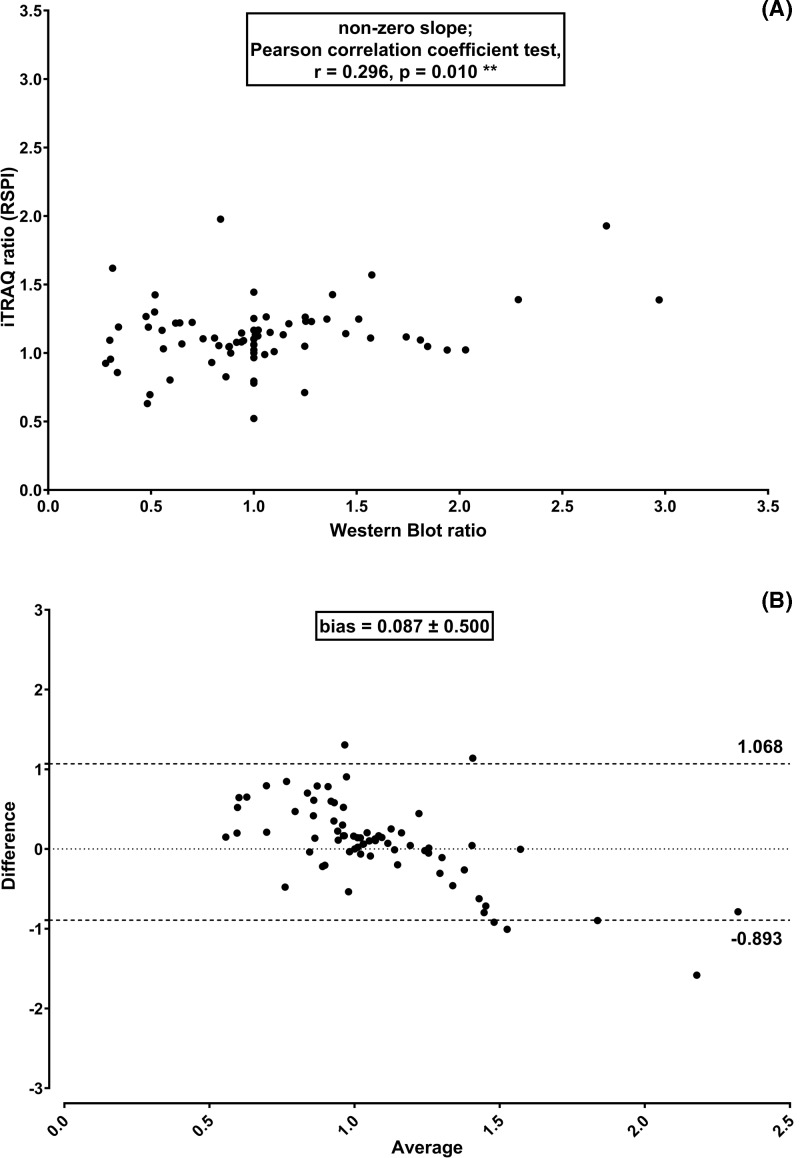
RPA-based quantitation strategy led to significant iTRAQ quantitation bias Linear regression plus Pearson correlation coefficient test (**A**) and Bland–Altman comparison plot (**B**) to assess correlation and agreement between iTRAQ based on RPA and Western blot protein quantitation along five independent experiments (biological replicates).

iTRAQ quantitation using the commercial method based on RPA displayed no significant linear correlation with Western blot quantitation (zero-slope; Pearson correlation coefficient r = −0.074, *P*=0.526) (Figure 2A). Furthermore, the average difference between Paragon – ProteinPilot – CiR-C and Western blot methods was −0.275 ± 0.961 (approx. 30% of a given iTRAQ ratio) while 95% Limits of Agreement were −2.163 and 1.609 (Figure 2B). Auto bias correction did improve neither accuracy nor precision as it worsened ratio compression and non-correlation to Western blot (zero-slope; Pearson correlation coefficient r = 0.072, *P*=0.537) and the average bias increased (−0.384 ± 0.731) even though 95% Limits of Agreement were tighter (−1.816 and 1.049) (data not shown). Moreover, the RPA method resulted in several mismatches where Western blot could not be compared with iTRAQ because of missing iTRAQ quantitation (iTRAQ ratio = 0).

**Figure 3:**
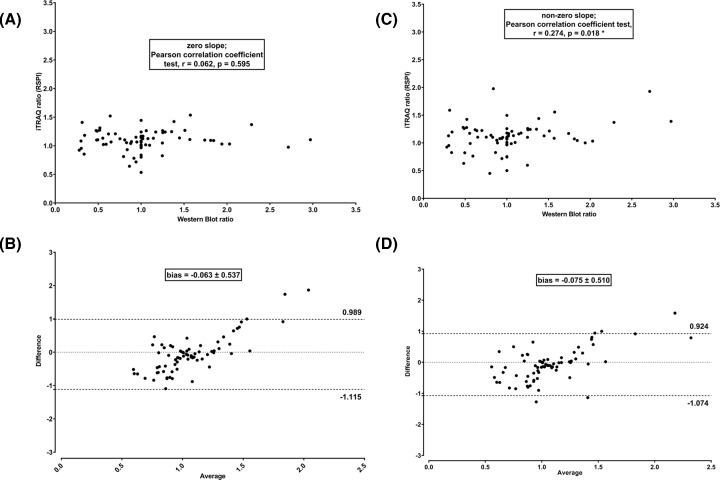
RSPI-based quantitation strategy led to unbiased, reliable iTRAQ quantitation Linear regression plus Pearson correlation coefficient test (**A**) and Bland–Altman comparison plot (**B**) to assess correlation and agreement between iTRAQ based on RSPI and Western blot protein quantitation along five independent experiments (biological replicates).

Concerning the quantitative results obtained from the Mascot Server – jTRAQx – CiR-C strategy based on RSPI, a statistically significant linear association with Western blot quantitation was observed (non-zero slope; Pearson correlation coefficient r = 0.296, *P*=0.010**) (Figure 3A). The average difference was 0.087 ± 0.500 (approx. 9% of a given ratio). The 95% Limits of Agreement were closer, i.e., −0.893 and 1.068 (Figure 3B).

In parallel, the two data processing strategies were applied to the analysis of MS/MS data of UPS1 spiked-in, iTRAQ-labeled LLC PK-1 protein extracts, for MS-related benchmarking (Supplementary Figure S1).

The Paragon – ProteinPilot – CiR-C data processing pipeline resulted in the monitoring of 24 out of 48 UPS1 proteins (Supplementary Figure S1B). No significant differences (One-way ANOVA, *P*=0.3532) were observed between DOWN:N1, UP:N1 and N2:N1 ratios. The global expression profile failed to reflect the differential spike-in of UPS1 proteins. Ratio compression towards ratio = 1.00 was observed.

The Mascot Server – jTRAQx – CiR-C data processing pipeline resulted in the monitoring of 20 out of 48 UPS1 proteins (Supplementary Figure S1C). DOWN:N1, UP:N1 and N2:N1 ratios were significantly different (One-way ANOVA, *P*<0.0001***). N2:N1 ratios were around ratio = 1.00 (mean = 1.01 ± 0.01). DOWN:N1 ratios were significantly lower than N2:N1 ratios (Dunnett’s post-test, mean = 0.95 ± 0.02, *P*<0.01**), reflecting the 25% decrease in UPS1 protein abundance after the spike-in of less UPS1 proteins. UP:N1 ratios were significantly higher than N2:N1 ratios (Dunnett’s post-test, mean = 1.07 ± 0.02, *P*<0.05*), reflecting the 25% increase in UPS1 protein abundance after the spike-in of more UPS1 proteins. The global expression profile successfully reflected the differential spike-in of UPS1 proteins. Nonetheless, ratio compression towards ratio = 1.00 was still observed.

### Refinement of the CiR-C algorithm

The CiR-C script uses two thresholds for peptide selection: a peptide significance threshold (given by the Mascot score) and a peptide frequency threshold (defined as the minimum number of experiments in which a given peptide must be identified and quantified). To establish the best compromise between reliability of iTRAQ quantitation and time/cost efficiency, we explored how threshold tuning may affect (strengthen or weaken) CiR-C results.

**Figure 4:**
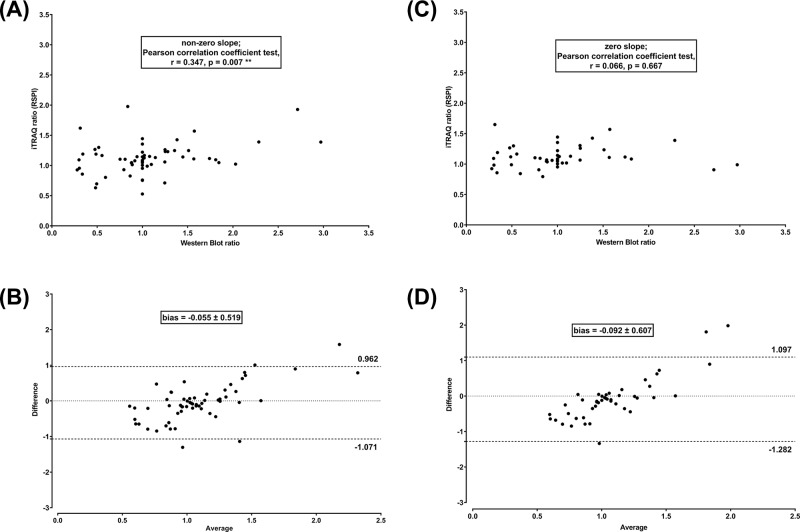
Tuning of Mascot score to more or less permissive thresholds did not improve iTRAQ quantitation performance Linear regression plus Pearson correlation coefficient test (**A** and **C**) and Bland–Altman comparison (**B** and **D**) to assess the correlation and agreement between RSPI and Western blot ratios in five independent experiments (biological replicates) after adjustment to more (A and B) or less (C and D) permissive Mascot score.

The RSPI calculated after tuning the first threshold to either a permissive (Mascot score > 20) or a stringent (Mascot score > 40) cut-off value were compared with Western blot standards (Figure 4). When opening to more peptides (lower Mascot score) the linear correlation was lost (zero slope; Pearson correlation coefficient test, r = 0.062, *P*=0.595) (Figure 4A); doing so, the mean difference compared with Western blot was slightly reduced (−0.063 ± 0.537, 95% Limits of Agreement 0.989 and −1.115) (Figure 4B). When restricting to less peptides (higher Mascot score) the linear correlation was not improved (non-zero slope; Pearson correlation coefficient r = 0.274, *P*=0.018*) (Figure 4C); again, the difference compared with Western blot was slightly reduced (−0.075 ± 0.510, 95% Limits of Agreement 0.924 and −1.074) (Figure 4D).

**Figure 5:**
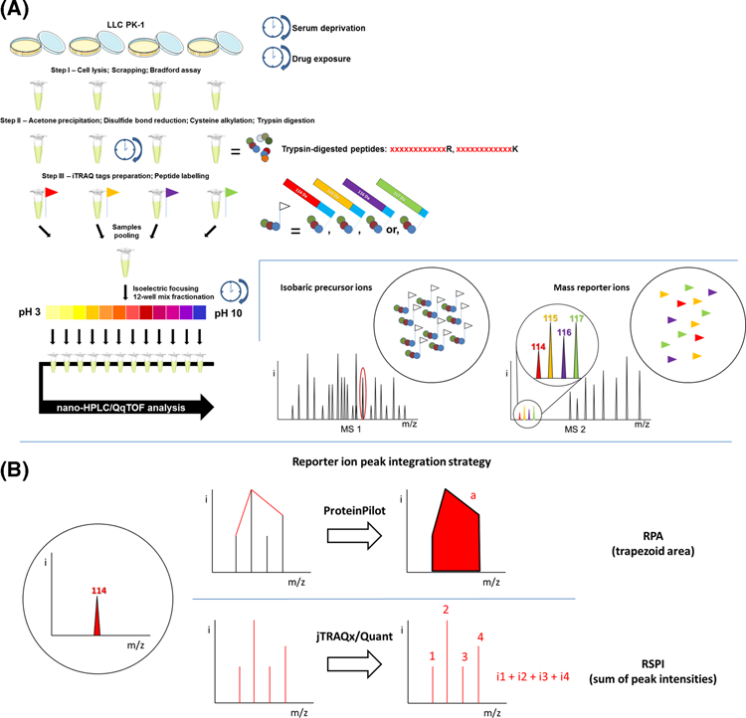
Peptide occurence in, at least, four biological replicates was mandatory for reliable iTRAQ quantitation by the Mascot-jTRAQ-CiR-C strategy Linear regression plus Pearson correlation coefficient test (**A** and **C**) and Bland–Altman comparison plot (**B** and **D**) to assess correlation and agreement between RSPI and Western blot ratios along 4 (A and B) or 3 (C and D) independent experiments (biological replicates).

The RSPI computed after adjustment of peptide frequency threshold to lower levels were plotted against Western blot results (Figure 5). Using peptides from four out of five independent experiments (biological replicates) resulted in a significant improvement of correlation (non-zero slope; Pearson correlation coefficient r = 0.347, *P*=0.007**) (Figure 5A) and a greater difference compared with Western blot (−0.055 ± 0.519, 95% Limits of Agreement 0.962 and −1.071) (Figure 5B). Conversely, using peptides from only three out of five independent experiments (biological replicates) led to a loss of correlation (zero slope; Pearson correlation coefficient r = 0.066, *P*=0.667) (Figure 5C) and a greater difference compared with Western blot (−0.092 ± 0.607, 95% Limits of Agreement 1.097 and −1.282) (Figure 5D).

## Discussion and conclusion

Since its first description by Ross et al. in 2004, iTRAQ has been widely used for multiplexed analysis of proteomes. The advantages and drawbacks of iTRAQ have been widely addressed in the literature, following the many developments of technical optimizations, analysis strategies and tools to improve quantitation precision and accuracy [24–27]. However, there is still no consensual technique in sample preparation and analysis or data processing.

**Scheme 2:**
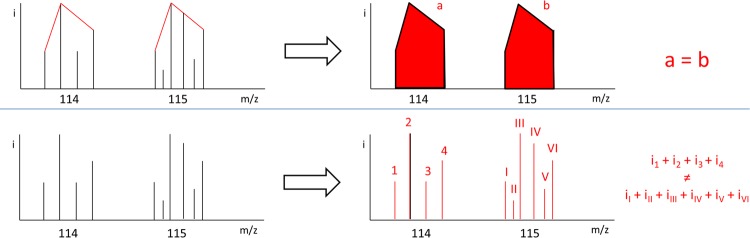
RSPI fit better than RPA to report tag signature ion counts

iTRAQ results in rich and complex MS/MS datasets which require thorough processing and solid statistics to reach relevant conclusions. In this respect, the choice of a tag ratio calculation method is crucial. Two potential strategies have been proposed: either RPA or RSPI. The two methods are not equivalent since peak area measurement suffers from a major bias originated from the way reporter ion signals are processed, as described by Boehm et al. [16]. It is worth noting that peak intensities are proportional to reporter mass ion counts, whereas peak areas are not (Scheme 2). Therefore, the RSPI is more likely to provide a reliable rendering of the actual ion count detected by mass spectrometer. Numerous studies reported the necessity of RPSI-based quantitation workflows to obtain robust, precise, accurate and sensitive when using high resolution MS platforms (Orbitraps and TripleTOF 5600) [28–30] We confirmed the superiority of RSPI since the commercially available RPA strategy failed to report significant biological variations assessed by Western blot analysis of LLC-PK-1 protein extracts and iTRAQ-nanoLC–MS/MS benchmark analysis of UPS1 spiked-in LLC-PK-1 protein extracts. In contrast, with the lowest bias and best correlation, the RSPI from Mascot – jTRAQx – CiR-C data processing strategy provided the most reliable set of quantitation ratios when compared with Western blot performance. As compared with the Western blot ‘quality control’, the median RPA showed dramatic differences leading to a global misrepresentation of changes in protein expression. Conversely, the median RSPI for the set of proteins of interest were well correlated and showed moderate and unbiased differences compared with Western blot. Similar observations were made monitoring the protein abundance of spiked-in UPS1 proteins after differential spike-in.

Western blot was chosen as a non-MS-based quality control for iTRAQ because the technique has already been successfully used to validate iTRAQ. Either alone [31,32], in parallel with other classical molecular biology techniques such as RT-qPCR [33,34], or, in parallel with MS-based targeted methods like selected reaction monitoring/multiple reaction monitoring (SRM/MRM) [35,36], Western blot always managed to validate and confirm results from both iTRAQ and other, more performant, validation techniques. Most importantly, the technique was the first to assess performance issues when discrepancies between Western blot and iTRAQ results highlight ratio compression and underestimation [37]. The observed difference between iTRAQ ratios and Western blot ratios may be explained by the intrinsic ratio compression due to background noise at the low m/z end, by co-elution of peptides with close m/z, and to a lesser extent by tag purity and inter-contamination [38]. Even if correction factors are provided to take tag ‘impurity’ into account, the background mass spectrometry noise brings the ratio towards unity. However, the custom-made strategy using the RSPI appeared to minimize these differences with respect to the commercially available strategy.

Differences between iTRAQ and Western blot may also be explained by the technical evolution of LC–MS-based quantitative proteomics when compared with antibody-based approaches. On one hand, Western blot remains a qualitative and semi-quantitative technique with an inherent variability in analytical sensitivity, specificity and reproducibility, especially because of the multifactorial (e.g. variability of antibodies specificity, non-linearity of chemiluminescent reaction) non-linearity between protein abundance and signal intensity [39,40]. On the other hand, the use of high-performance liquid chromatography (such as nanoLC) online with high-resolution mass spectrometers (such as TripleTOF 5600+ QqTOF mass spectrometer) has outperformed Western blot with greater sensitivity, selectivity, specificity and reproducibility, wider linear dynamic range, increased accuracy and precision, high-throughput of in-depth information [41–45]. Despite these considerations, Western blot quality control alone highlighted the superiority of the RSPI-based Mascot – jTRAQx – CiR-C pipeline over the RPA-based Paragon – ProteinPilot – CiR-C pipeline, later confirmed thanks to the UPS1 spike-in experiments.

A major point of iTRAQ data processing is the upstream tolerance for peptide characterization and identification. More peptides were identified with Mascot Server than with Paragon, mainly because mass tolerances were more stringent with the latter, due to the use of non-customizable manufacturer’s parameters optimized for data generated by TripleTOF 5600+ mass spectrometers. Mass tolerances with Mascot Server were chosen to be sufficiently permissive to provide enough data for further selection. The choice of stringent parameters for identification is in complete agreement with the RPA quantitation strategy, as implemented in ProteinPilot, where peak width is essential to reliable quantitation. Conversely, our approach postpones the application of stringent conditions to after quantitation.

Another major point of iTRAQ data processing is the downstream management of irrelevant data. It can be done manually but it is time-consuming and error-prone due to the huge amount of information at the peptide level as well as the number of replicates. Just like with every high-throughput approach, the analysis of iTRAQ results needs to be automated. The CiR-C algorithm was designed to be the simplest in terms of data elimination and transformation. Likely to exacerbate inherent issues – such as ratio-compressing variance-stabilizing normalization – heavy data transformation was not used after jTRAQx or ProteinPilot data processing. The biological significance of the results arose from: (i) a focus on peptide selection thanks to the probabilistic Mascot score or its Paragon counterpart (Confidence score); (ii) the restriction to selected peptides based on their occurrence among multiple biological replicates; (iii) the calculation of median and weighted mean ratios for each set of peptides obtained from a given protein. As mentioned above, upstream data processing was willingly permissive to sustain this statistical approach. It resulted in less loss of information and good data fitting. Paradoxically, stringent identification criteria also resulted in increased output, including a large amount of irrelevant data: when parsing peptide summaries, it appeared that irrelevant data were essentially null ratios, i.e., the technical impossibility to provide peptide quantitation from the MS2 mass spectra.

The confidence threshold for the Mascot score was a compromise between the generation of aberrant information and the loss of information. This threshold highly depends on the number of peptides characterized by mass spectrometry. The probability of a random match was first set to 1 out of 1000 (s = 30, *P*=0.001) and the test of scores 10-fold apart from our initial choice (s = 20, *P*=0.01) confirmed how close it is from a potential optimum for the size of our datasets. Indeed, this threshold highly depends on the number of peptides characterized in MS/MS.

The occurrence threshold was set to the largest possible values – the more peptides, the better – hence the restriction to ubiquitous peptides, i.e., peptides retrieved from all five biological replicates. The main source of variations when using iTRAQ is of biological origin (±25%) [46]. The number of biological replicates was set to five independent experiments (biological replicates), corresponding to the use of a complete given set of iTRAQ tags. However, as a matter of time and cost efficiency, the possibility to reduce the number of independent experiments (biological replicates) had to be addressed. As expected, considering biological variations are the most impactful source of variability of iTRAQ, we showed that using four independent replicates is the lowest limit to obtain reliable results, five replicates appearing optimal under our experimental conditions.

In summary, this work demonstrated that RSPI outperform the commercially available RPA in quantifying biological modifications using iTRAQ. Furthermore, we propose a Mascot – jTRAQx – CiR-C strategy as a simple yet powerful answer to the need for an all-inclusive suite for iTRAQ data processing.

## Significance of the study

In this work, an in-house algorithm named Customizable iTRAQ Ratio Calculator (CiR-C) was implemented to process large datasets and compute final quantitation (median, weighted mean and standard deviation) for iTRAQ-based shotgun proteomics. This algorithm was used to retreat datasets in the comparison between two workflows based on the two strategies of MS/MS signal integration (RPA versus RSPI) for iTRAQ quantitation in the perspective of the proteome monitoring of tubular proximal cell lysates. RSPI was confirmed to be the best-suited strategy when using high-resolution MS platforms. The RSPI-based iTRAQ workflow happened to allow reliable and robust protein expression measurement. CiR-C proved to be a promising, simple and powerful, adjunct to iTRAQ data processing.

## Supporting information

**Supplementary Figure S1:** 

## Abbreviations

ACN: acetonitrile
CiR-C: Customizable iTRAQ Ratio Calculator
CNI: CalciNeurin Inhibitor
CsA: Cyclosporine A
FA: Formic Acid
ICAT: Isotope-Coded Affinity Tags
IEF: IsoElectric Focusing
IPG: immobilized pH gradient
iTRAQ: isobaric Tags for Relative and Absolute Quantitation
HEPES: HEPES buffer
LFQ: Label-Free Quantification
LLC PK-1: Lilly Laboratories Cells Porcine Kidney 1
MS/MS: tandem mass spectrometry
m/z: mass-to-charge ratio
nano-HPLC: nano-scale High-Performance Liquid Chromatography
NFAT: Nuclear Factor of Activated T cells
Q-Q-TOF: Quadrupole-Quadrupole-Time-Of-Flight
RIPA: RadioImmunoPrecipitation Assay buffer
RPA: Ratio of Peak Areas
RSPI: Ratio of the Sums of Peak Intensities
SILAC: Stable Isotope Labeling of Amino acids in Cell culture
Tac: Tacrolimus
TBS-T: Tris-Buffered Saline-Tween 20
UPS1: Universal Protein Standard mixture 1
