# Supplementary material for: Sum of peak intensities outperforms peak area integration in iTRAQ protein expression measurement by LC-MS/MS using a TripleTOF 5600+ platform
Source: Biosci Rep. 2019 Jun 7;39(6):BSR20190904. doi: 10.1042/BSR20190904 (PMC6554230; doi:10.1042/BSR20190904)

A

Sample	Benchmark Set	UPS 1 : protein ratio	4-plex distribution
LLC PK-1	1	1:15 (UP)	114
		1:25 (DOWN)	115
		1:20 (N1)	116
		1:20 (N2)	117
	2	1:15 (UP)	115
		1:25 (DOWN)	116
		1:20 (N1)	117
		1:20 (N2)	114
	3	1:15 (UP)	116
		1:25 (DOWN)	117
		1:20 (N1)	114
		1:20 (N2)	115

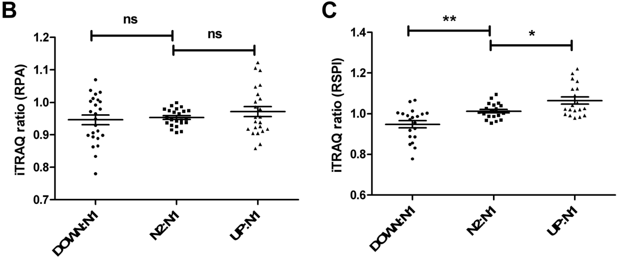

Supplement: Supplementary file 1 [file bsr20190904_Supp1.pdf]
